# *Streptomyces* RNases – Function and impact on antibiotic synthesis

**DOI:** 10.3389/fmicb.2023.1096228

**Published:** 2023-04-11

**Authors:** George H. Jones

**Affiliations:** Department of Biology, College of Arts and Sciences, Emory University, Atlanta, GA, United States

**Keywords:** *Streptomyces*, antibiotic, regulation, ribonuclease, RNA decay, RNA processing

## Abstract

*Streptomyces* are soil dwelling bacteria that are notable for their ability to sporulate and to produce antibiotics and other secondary metabolites. Antibiotic biosynthesis is controlled by a variety of complex regulatory networks, involving activators, repressors, signaling molecules and other regulatory elements. One group of enzymes that affects antibiotic synthesis in *Streptomyces* is the ribonucleases. In this review, the function of five ribonucleases, RNase E, RNase J, polynucleotide phosphorylase, RNase III and oligoribonuclease, and their impact on antibiotic production will be discussed. Mechanisms for the effects of RNase action on antibiotic synthesis are proposed.

## Introduction

Members of the genus *Streptomyces* are Gram-positive, soil dwelling bacteria notable for their ability to undergo morphological differentiation (sporulation) and for their production of antibiotics (Chater, 2016; Hopwood, 2019). Both sporulation and antibiotic biosynthesis are tightly and elegantly regulated. A recent review has posited four levels of the regulatory cascade that controls *Streptomyces* antibiotic synthesis: (1) signals that initiate antibiotic synthesis; (2) global regulators of antibiotic synthesis; (2) cluster-situated regulators CSRs, formerly referred to as pathway-specific regulators [PSRs] or SARPs [*Streptomyces* antibiotic regulatory proteins]; (4) feedback inhibition (Xia et al., 2020). These various levels of regulation involve the participation of signaling molecules such as the γ-butyrolactones and (p)ppGpp, activator and repressor proteins, two-component regulatory systems, RNA polymerase sigma factors and anti-sigma factors, regulatory RNAs and other regulatory molecules (Bibb, 1996; Liu et al., 2013; Niu et al., 2016).

One group of enzymes that affects antibiotic biosynthesis in *Streptomyces* is the RNases, enzymes that degrade RNAs either exo- or endonucleolytically (Jones, 2010). This review will focus on the roles of five specific RNases, their function in *Streptomyces* and their roles in the control of antibiotic production, *viz.* RNase E, RNase J, polynucleotide phosphorylase, RNase III and oligoribonuclease. Mechanisms to explain the effects of these enzymes on antibiotic production in *Streptomyces* are proposed. This subject was last reviewed in 2010 (Jones, 2010) and the current review will focus on experimental results obtained since that date and on their interpretation. In some cases, the analysis of more recent experiments will require referencing studies published prior to 2010.

## RNase E

RNase E is a single-strand specific endoribonuclease that is widely distributed in bacteria (Cohen and Mcdowall, 1997). RNase E from *Escherichia coli* is a 1,061 amino acid protein (Casaregola et al., 1992) that is organized as two domains, an N-terminal catalytic domain (NTH) and a C-terminal scaffold domain (CTH) (Mcdowall and Cohen, 1996; Mackie, 2013). The crystal structures of the RNase E catalytic domain (Callaghan et al., 2005) and of the RNase E apoprotein (Koslover et al., 2008) have been determined. These structural studies showed that the catalytic domain is comprised of several subdomains that are involved in catalysis, RNA binding and sensing the 5′-end of target RNA molecules (Koslover et al., 2008). The C-terminal scaffold domain of RNase E binds polynucleotide phosphorylase (PNPase), the RNA helicase, RhlB, and enolase (Py et al., 1996), to form the RNA-degrading complex known as the degradosome (Vanzo et al., 1998). RNase E is tethered to the cell inner membrane in *E. coli* (Khemici et al., 2008).

Hagege and Cohen demonstrated the presence of an RNase E-like activity in the model species for the study of streptomycetes, *Streptomyces coelicolor* (Hagege and Cohen, 1997). Lee and Cohen identified the gene encoding the enzyme responsible for that activity, *sco2599* (*rns*) and overexpressed and characterized the gene product, designating it RNase ES (Lee and Cohen, 2003). *S. coelicolor* RNase ES is even larger that RNase E with 1,340 amino acids (Lee and Cohen, 2003). The RNase E and RNase ES catalytic domains are 33% identical and 50% similar in amino acid sequence. Key residues involved in catalysis at the active site, in sensing the 5′-end of target RNA molecules and in Zn ion binding (Callaghan et al., 2004; Koslover et al., 2008) are strongly conserved between the two enzymes (Table 1).

**Table 1:** Conserved amino acids in the domains of *E. coli* (Ec) RNase E and *S. coelicolor* (Sc) RNase ES.

Catalytic site	Ec: F57, K112, D303, N305, D346
	Sc: F619, K671, D862, N864, D898
5’-sensing domain	Ec: G124, V128, R169, T170
	Sc: G683, V687, R726, T727
Zinc binding motif	Ec: CprCsGtG
	Sc: CvrCnGrG

Unlike RNase E, the catalytic domain of RNase ES is located near the center rather than at the N-terminus of the enzyme (Lee and Cohen, 2003). Lee and Cohen provided evidence indicating that regions near the N- and C-termini of RNase ES interact with polynucleotide phosphorylase. Western blots of immunoprecipitates obtained by treating mycelial extracts from *S. coelicolor* with antibody to RNase ES revealed a co-precipitated protein that reacted with antibody to PNPase from *Streptomyces antibioticus* (Jones, 1994). Western blotting experiments utilizing truncated forms of RNase ES, lacking *ca.* 800 residues from the N-terminal end or *ca.* 400 residues from the C-terminal end of full length RNase ES, indicated that sequences interacting with PNPase were present in both the N- and C-terminal regions of RNase ES. Lee and Cohen argued that the N- and C-terminal regions of RNase ES are functionally equivalent to the C-terminal scaffold domain of RNase E. The authors further speculated that *S. coelicolor* might contain a macromolecular complex similar to the degradosome (Lee and Cohen, 2003), but no additional evidence supporting this suggestion has been adduced. It should also be noted that a subsequent study, Yeom et al. were unable to demonstrate the interaction of *E. coli* PNPase with RNase ES (Yeom et al., 2008). The structural basis for the discrimination by RNase ES between *E. coli* and *S. antibioticus* PNPases remains to be determined.

RNase ES activity increased as *S. coelicolor* mycelium progressed from exponential growth to stationary phase in liquid cultures and from mycelial growth to spore formation on solid media (Hagege and Cohen, 1997). RNase E is essential in *E. coli* (Apirion and Lassar, 1978) but the corresponding gene (*sco2599*, *rns*) could be disrupted in *S. coelicolor* with only minimal impact on the physiology of the null mutant (Lee and Cohen, 2003). Lee and Cohen noted only a 10% decrease in the growth rate of the *rns* null mutant as compared with the parental strain.

The effects of the *rns* null mutation on the transcriptome of *S. coelicolor* have not been examined, thus not much is known about the role of RNase ES in RNA processing and degradation. In *in vitro* studies, Hagege and Cohen and Lee and Cohen demonstrated RNase ES cleavage of RNA I, an antisense repressor of the replication of ColE1-type plasmids in *E. coli* (Hagege and Cohen, 1997; Lee and Cohen, 2003). RNase ES cleaved that RNA at the same site as did RNase E. RNase E and ES also cleaved the *E. coli* 9S RNA precursor of 5S ribosomal RNA. However, the cleavage sites recognized by the two enzymes were not identical. RNase ES correctly processed the *E. coli* pM1 RNA, the precursor of the RNA component of RNase P (Inagawa et al., 2003).

Perhaps the most significant observation regarding RNase ES function is that the enzyme can substitute for RNase E in an *E. coli rne* (RNase E) null mutant. Lee and Cohen demonstrated that the *rne* null mutant when complemented by *rns* grew at rates that were comparable to the wild type strain. They showed further that the truncated RNase ES derivatives described above complemented the *rne* null mutation, since those derivatives contained the RNase ES catalytic domain (Lee and Cohen, 2003). Inagawa et al. confirmed the observation that *rns* complemented the *rne* null mutation and demonstrated further that both the 9 s rRNA precursor and the pM1 RNA were processed normally in the complemented mutant. These authors also showed that *rns* complemented a null mutation in the gene for RNase G, a paralog of RNase E (Inagawa et al., 2003). Taken together these observations suggest that RNase E and RNase ES play similar roles in their respective hosts. It is likely, therefore, that as with RNase E (Mackie, 2013), RNase ES is involved in mRNA degradation, ribosomal RNA processing, tRNA processing and the processing of small regulatory RNAs, among other likely functions. *S. coelicolor* contains RNase P (Kim et al., 2000) so it is possible that RNase ES is involved in processing the RNA component of that ribozyme.

The effects of the *rns* null mutation on antibiotic production by *S. coelicolor* were not examined in the studies of the Cohen group. Comparative studies in *E. coli* and *S. coelicolor* may bear on that issue. Lee et al. and Gao et al. characterized two proteins that modulate the activity of RNase E in *E. coli* (Lee et al., 2003; Gao et al., 2006). These proteins were designated RraA and RraB (Rra = Regulator of ribonuclease activity). RraA and RraB inhibit the activity of RNase E *in vivo* and *in vitro*, albeit by different mechanisms. RraA inhibits the enzyme by interacting with the NTH catalytic domain. Although inhibition of the catalytic activity of the isolated NTH by RraA could be demonstrated, that inhibition was enhanced by the presence of the scaffold domain (Lee et al., 2003; Gao et al., 2006). RraB binds to the RNase E scaffold domain and Gao et al. identified a specific region of the CTH that is essential for RraB binding (Gao et al., 2006). Both RraA and RraB inhibited the processing of the RNase P pM1 RNA *in vivo* and *in vitro*, although to different extents (Gao et al., 2006). An interesting property of both RraA and RraB is their ability to remodel the *E. coli* degradosome, changing the ratios of RNase E, PNPase, RhlB and enolase in that complex (Gao et al., 2006).

Yeom et al. (2008) obtained the interesting result that RraA and RraB from *E. coli* can inhibit the activity of RNase ES. This observation suggested that *S. coelicolor* might also contain proteins capable of regulating the activity of RNase ES in *trans*. Two such proteins have been found to date and were designated RraAS1 and RraAS2 (Ahn et al., 2008). RraAS1 is encoded by *sco5940* and RraAS2 by *sco7163*. The two proteins are *ca.* 25 and 17% identical to RraA, respectively, and are *ca.* 35 and 27% similar to RraA. They are less identical and similar to RraB. RraAS1 and S2 are also *ca.* 60 amino acids longer than RraA and B.

Heo et al. demonstrated that an *E. coli rne* null mutant expressing *rns* instead grew more slowly when RraAS2 was overexpressed in the mutant strain than in the absence of RraAS2 overexpression (Heo et al., 2016). Moreover, RraAS2 inhibited RNase ES cleavage of a truncated form of the pM1 RNA *in vitro* (Heo et al., 2016). Thus, as with RraA and B, RraAS2 is an inhibitor of RNase ES activity. Immunoprecipitation experiments, using extracts of *E. coli* cells expressing RraAS2 showed that, unlike RraA and B, RraAS2 did not affect the ratios of the enzymes contained in the *E. coli* degradosome (Yeom et al., 2008). Instead, RraAS2 decreased the affinity of RNase ES for its RNA substrates. Immunoprecipitation experiments demonstrated that RraAS2 binds directly to RNase ES and that this binding requires the N- and C- terminal scaffold domains of RNase ES. Truncated RNase ES derivatives lacking those domains did not bind RraAS2 (Heo et al., 2016).

Seo et al. (2017) studied the function of RraAS1 and its role in the physiology of *S. coelicolor*. RraAS1 overexpression increased the copy number of a ColE1-type plasmid in the *E. coli rne* null mutant expressing *rns*, presumably reflecting the inhibition of RNase ES by RraS1 thereby decreasing the levels of RNA I. Moreover, RraAS1 inhibited the RNase ES-catalyzed *in vitro* cleavage of a model substrate, a synthetic RNA containing the *E. coli* RNA I cleavage site recognized by RNase E. As with RraAS2, RraAS1 co-precipitated with RNase ES when extracts of *E. coli* cells expressing both proteins were treated with antibody to Myc-tagged RraAS1. Unlike the situation with RraAS2, truncated derivatives of RraAS1 lacking the N- and/or C-terminal scaffold domains were as functional as the full-length protein in inhibiting RNase ES activity *in vivo* and *in vitro*. Moreover, truncated RNase ES derivatives, lacking the N- and/or C-terminal scaffold domains, co-immunoprecipitated with RraAS1. These observations indicate that while RraAS2 interacts with the N- and C-terminal scaffold domains of RNase ES, RraAS1 interacts with the catalytic domain (Seo et al., 2017).

**Figure 1:**
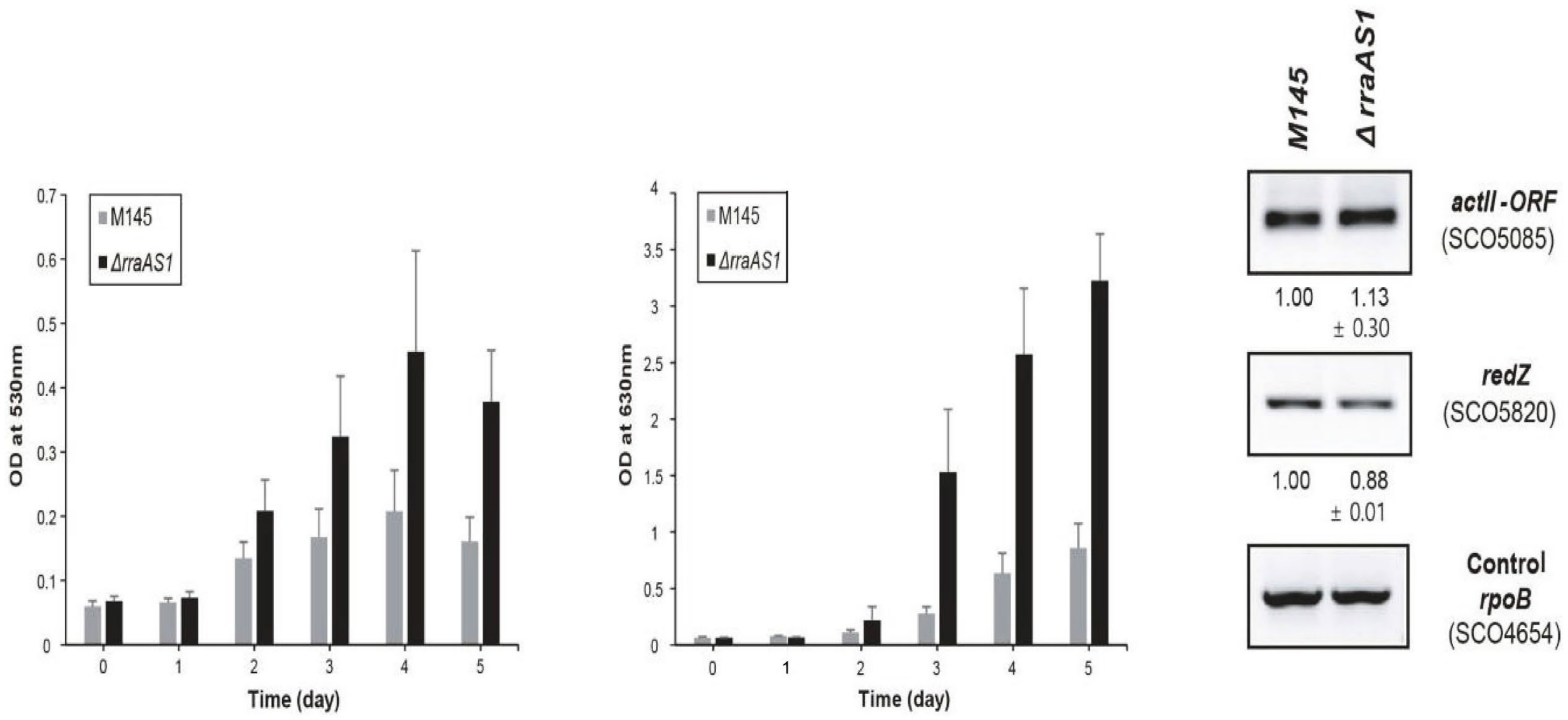
Overproduction of red (right bar graph) and act (left bar graph) by an *rraAS1* null mutant of *S. coelicolor*. The antibiotics were measured using spectroscopic methods from mycelium harvested at the times indicated on the x-axis of the figures. The antibiotic concentrations were normalized relative to mycelial dry weights. The rightmost part of the figure shows the results of an RT-PCR analysis of the levels of the CSRs, *actII-orf4* and *redZ* in the parental and *rraAS1* null mutant strains of *S. coelicolor*. Reprinted with permission from Seo et al. (2017).

What then is the connection between these analyses and antibiotic production by *S. coelicolor*? On laboratory growth media, wild type *S. coelicolor* produces four antibiotics, two pigmented ones, actinorhodin (act) (Cole et al., 1987) and undecylprodigiosin (red) (Feitelson et al., 1985) and two unpigmented ones, calcium dependent antibiotic (cda) (Hopwood and Wright, 1983) and methylenomycin (mmy) (Wright and Hopwood, 1976). Seo et al. (2017) constructed an *rraAS1* null mutant of *S. coelicolor* and examined the effects of the null mutation on the physiology of the organism. They observed a slightly increased growth rate of the mutant as compared with the parental strain. They also observed that both act and red were overproduced in the *rraAS1* null mutant. Overproduction began during vegetative growth of the mutant strain and continued for several days post-inoculation (Figure 1). At their maxima, red was produced at a level *ca.* twice that of the parental strain and act at almost four times the level observed for the parental strain (Figure 1). The authors examined the transcripts for two of the CSRs involved in act and red production, *viz. actII-orf4* and *redZ*. The levels of those transcripts were essentially identical in the *rraAS1* null mutant and the parental strain (Seo et al., 2017). It should be noted that the authors only examined CSR transcript levels at a single time point, mid-log phase. It is possible that levels for these transcripts did vary at other times during the growth of the organisms. Taken at face value, though, the results suggest that RraAS1 exerts its effect on act and red production at a level other than the CSRs. That level might be RNase ES.

## RNase J

“The lack of a bacterial 5′-to-3′-exonuclease was the accepted dogma, even for years after bacterial genomes sequences began to appear in the mid-1990s (Bechhofer and Deutscher, 2019).” That dogma was shown to be erroneous by the discovery in 2007 of two RNases (RNase J1 and J2) with 5′-3′-exonuclease activity in *Bacillus subtilis* (Mathy et al., 2007). These two enzymes were first identified in *B. subtilis* in 2005 (Even et al., 2005), but their exonuclease activities were not demonstrated until 2007 (Mathy et al., 2007). RNase J1 and J2 are paralogs, and they not only possess 5′-3′-exonuclease activity, they are bifunctional enzymes possessing endonuclease activities as well (Even et al., 2005; Mathy et al., 2007). RNase J1 has been shown to function in mRNA turnover, rRNA processing and in the processing of small RNAs in *B. subtilis* (Condon, 2010; Bechhofer and Deutscher, 2019). The role of RNase J2 remains somewhat controversial, at least in *B. subtilis*, since an *rnjB* (RNase J2) null mutant grew normally under laboratory conditions (Mader et al., 2008), while an *rnjA* (RNase J1) null mutant grew poorly (Figaro et al., 2013). RNase J1 and J2 can form heteromeric complexes in *B. subtilis* but the biological significance of this association remains unclear (Newman et al., 2011).

Since the discovery of RNases J1 and J2 in *B. subtilis*, orthologs have been identified in a number of other organisms, including Gram-negative bacteria, Archaea, and plant chloroplasts (Even et al., 2005). RNases J1 and J2 have been shown to be involved in a variety of biological processes in addition to RNA degradation and processing, including the conferral of multidrug resistance (Martini et al., 2022), controlling plasmid copy number (Guimaraes et al., 2021) and the modulation of cell morphology, primary metabolism and virulence (Luong et al., 2021).

RNase J was identified in *S. coelicolor* by Bralley et al. (2014a). Unlike *B. subtilis* (but like several other systems), *S. coelicolor* contains only one RNase J ortholog. *S. coelicolor* RNase J is *ca.* 38% identical and 60% similar to RNases J1 and J2 from *B. subtilis*. *S. coelicolor* RNase J was overexpressed and purified and its activities were examined using a model substrate, the *thrS* RNA, derived from the leader region of the threonyl-tRNA synthetase gene of *B. subtilis* (Condon et al., 1996). The *thrS* RNA was chosen to allow comparison of the results obtained with *S. coelicolor* RNase J with those of other studies using *thrS* RNA (Even et al., 2005). Bralley et al. (2014a) demonstrated that *S. coelicolor* RNase J possessed both 5′-3′-exonuclease and endonuclease activities. As was observed in studies with other RNases J, the *S. coelicolor* enzyme preferred a 5′-monophosphorylated substrate to a 5′-triphosphorylated substrate. Thus, the *S. coelicolor* enzyme did not produce GTP when challenged with 5′-triphosphorylated *thrS* RNA with G as the 5’terminal base and instead generated a series of oligonucleotides 2–10 residues in length. In studies with RNase J from *Mycobacterium smegmatis*, Taverniti et al. (2011) showed that the enzyme did produce GTP from the 5′-triphosphorylated *thrS* substrate. These differences relate to the mechanisms proposed for the exo- and endoribonuclease activities of RNase J and will be considered in detail below.

The crystal structure of *S. coelicolor* RNase J was determined by Pei et al. (2015) and consideration of that structure suggests a mechanism for exonuclease cleavage and for the preference for monophosphorylated substrates. In the model proposed by Pei et al. to describe substrate interactions for exo- and endonuclease cleavages by *S. coelicolor* RNase J, an RNA substrate is bound at the catalytic site adjacent to which is a binding pocket which can accommodate a 5′-monophosphorylated end. Binding of that 5′-end then leads to exonuclease cleavage of the substrate at the active site, producing a mononucleotide and a new 5′-monophosphorylated end that can be further processed by the enzyme. A triphosphorylated 5′-end cannot be accommodated by the binding pocket, the RNA substrate slides through the active site in consequence, and oligonucleotides are produced by endonuclease cleavage (Pei et al., 2015). This model may also explain the results described by Taverniti et al. (2011), who suggested that the substrate binding pocket of *M. smegmatis* RNase J was unable to accommodate a 5′-triphosphorylated substrate, that the substrate RNA slid past the active site and that GTP was released by an endonucleolytic cleavage. If this model is correct, it is interesting that *M. smegmatis* RNase J could release the 5’-GTP, but *S. coelicolor* RNase J could not.

**Figure 2:**
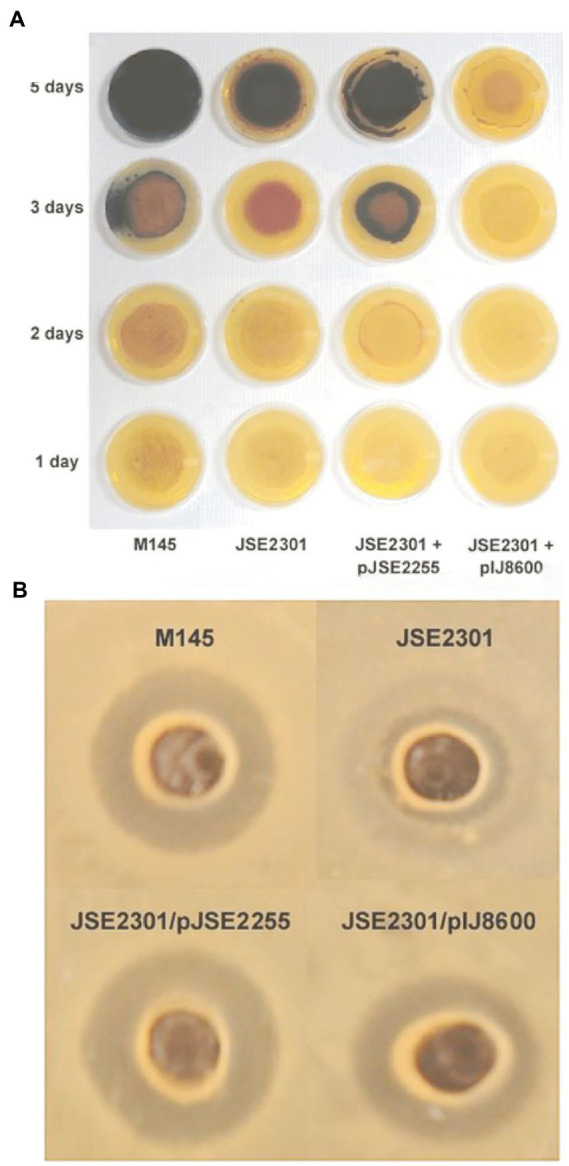
Effects of an *rnj* null mutation on antibiotic production in *S. coelicolor*. The top panel **(A)** shows the temporal progress of act and red production in the parental (M145) and mutant (JSE2301) strains cultured on solid medium that supports antibiotic biosynthesis. pJSE2255 is a plasmid containing the wild type RNase J gene cloned into the streptomycete expression vector, pIJ8600. In the *rnj* null mutant, act production is suppressed while red is overproduced. The bottom panel **(B)** shows the levels of cda production in the wild type and parental *S. coelicolor* strains. The figure shows zones of inhibition produced by cda action against a cda-sensitive bacterial strain, *B. subtilis* BG267. Reprinted with permission from Pei et al. (2015).

RNase J is not essential in *S. coelicolor* and Bralley et al. (2014a). were able to construct an *rnj* null mutant. The authors reported no obvious differences in the growth of the null mutant on solid or liquid media as compared with the parental strain nor in its ability to sporulate. Bralley et al. observed that the *rnj* null mutant showed significantly altered capacities to produce antibiotics as compared with the parent. In particular, act production was delayed compared to the parental strain (Figure 2). Act was observed after 3 days of growth on production medium in the parent but no act was observed at that time point in the null mutant. Production of cda was significantly diminished in the null mutant as compared with the parental strain (Figure 2). The *rnj* null mutation led to substantial overproduction of the red antibiotic (Bralley et al., 2014a). The authors did not examine the effects of the mutation on the CSRs that are involved in regulating production of act, red and cda.

Jones et al. (2014) examined that role of RNase J in another streptomycete, *Streptomyces venezuelae*. As with *S. coelicolor*, *rnj* is not essential in *S. venezuelae* but unlike *S. coelicolor*, the *rnj* null mutation affected strain morphology. Jones et al. observed several sporulation defects in the *rnj* null mutant. They reported that mutant spores were unpigmented while wild types spores were associated with a green pigment. In liquid medium, Jones et al. found that the onset of sporulation was significantly delayed as compared with the wild type, that fewer spore chains were formed by sporulating cultures of the *rnj* mutant as compared with the wild type strain and that the mutant spores were shorter and more sensitive to heat than wild type. Jones et al. examined ribosome profiles in the *rnj* null mutant and reported that the mutant ribosome population contained a significant fraction of ribosome dimers, sedimenting at *ca.* 100S in sucrose gradients. The authors did not examine the ability of the *rnj* mutant to synthesize proteins *in vivo* or *in vitro*, either in terms of overall amino acid incorporation or by looking at the synthesis of specific gene products.

Jones et al. found that the *rnj* mutation led to changes in the antibiotic sensitivity of mutant strains, leading to increased sensitivity to some antibiotics and decreased sensitivity to others (Jones et al., 2014). They examined the effects of the *rnj* mutation on the production of the antibiotic jadomycin by *S. venezuelae.* They observed a 20–30% decrease in jadomycin production as compared to the wild type strain but no difference in the transcript levels of a CSR, *jadJ*, or two biosynthetic genes, *jadA* an *jadM*, involved in jadomycin production in *S. venezuelae*.

RNase J function has also been studied recently in the lincomycin producing species, *Streptomyces lincolnensis*. Wang et al. observed that deletion of *rnj* led to a 22.4-fold increase in lincomycin production as compared with the wild type strain and that this increase was 85% of that previously observed in a commercial overproducing strain of *S. lincolnensis* obtained by traditional mutation and screening methods (Wang et al., 2020).

It seems clear from the foregoing analyses that RNase J plays a significant role in cellular economy in *Streptomyces* and in the production of streptomycete antibiotics. Further studies are required to illuminate the roles of RNase J in these processes.

## Polynucleotide phosphorylase

Polynucleotide phosphorylase (PNPase) is a 3′-5′-exoribonuclease that is found in bacteria, protists and in plant and animal organelles (Wright et al., 1977; Leszczyniecka et al., 2004). The PNPase reaction is as follows:


$${\left(p^{5'}N^{3'}\mathrm{OH}\right)}_{X}+\mathrm{Pi}⇆{\left(p^{5'}N^{3'}\mathrm{OH}\right)}_{X-1}+{\mathrm{pp}}^{5}N$$

where N is any of the four bases found in RNA. The forward reaction describes the phosphorolysis of RNA chains, in which the enzyme processively removes one residue at a time from the 3′-end, concomitantly generating nucleoside diphosphates (Grunberg-Manago and Ochoa, 1955; Singer, 1958; Godefroy-Colburn and Grunberg-Manago, 1972; Littauer and Soreq, 1982; Littauer, 2005). The reaction is fully reversible, however, and PNPase will synthesize polyribonucleotide chains *de novo* (polymerization) using nucleoside diphosphates as substrates, generating inorganic phosphate as a by-product (Grunberg-Manago and Ochoa, 1955; Grunberg-Manago et al., 1956; Godefroy-Colburn and Grunberg-Manago, 1972; Littauer and Soreq, 1982; Littauer, 2005).

*Streptomyces* PNPase has been characterized in terms of biochemical function and structure. Indeed, the first published PNPase crystal structure was that of the enzyme from *S. antibioticus* (Symmons et al., 2000). The enzyme was described as a trimer of dimers. The trimer designation derives from the observation that the native enzyme is composed of three PNPase monomers. Each monomer consists of two RNase PH domains which presumably arose by gene duplication and fusion, thus a trimer of dimers (Symmons et al., 2000). The enzyme also contains KH and S1 domains at its C-terminal end (Bycroft et al., 1997; Lewis et al., 2000). These domains have been shown to be involved in RNA binding and in the autoregulation of PNPase expression (see further below). In addition to the catalysis of phosphorolysis and polymerization, *Streptomyces* PNPases have several novel features including function as RNA 3′-polyribonucleotide (poly(A)) polymerases; stimulation of phosphorolysis by nucleoside diphosphates; and, inhibition of phosphorolysis and polymerization by ppGpp. These features have been recently reviewed (Jones, 2018) and will not be discussed in detail here (but see further below regarding the possible connection between ppGpp inhibition and antibiotic synthesis).

In *E. coli* and other bacteria, the PNPase gene, *pnp*, is transcribed as a part of an operon that also includes *rpsO*, the gene for ribosomal protein S15 (Régnier and Portier, 1986; Clarke and Dowds, 1994; Luttinger et al., 1996). In many of these organisms, the *rpsO*-*pnp* operon is transcribed from two promoters, one upstream of *rpsO* and one in the intergenic region between *rpsO* and *pnp* (Portier and Regnier, 1984). Thus, transcription of the operon produces an *rpsO* transcript and a readthrough transcript from P*rpsO*, and a *pnp* transcript from P*pnp*. The intergenic region also contains a stem-loop structure or hairpin which is involved in the regulation of expression of the operon (Régnier and Portier, 1986).

The organization of the *rpsO*-*pnp* operon in *Streptomyces* resembles that of *E. coli* and other bacteria but there is at least one important difference. While the operon is transcribed from two promoters in *E. coli*, *rpsO*-*pnp* is transcribed from four promoters in *S. coelicolor*, two upstream of *rpsO*, P*rpsO* A and B, and two in the intergenic region, P*pnp* A and B (Bralley et al., 2014b). As in *E. coli*, an *rpsO* transcript, a readthrough transcript and a *pnp* transcript are produced from the operon (Gatewood et al., 2011). These four promoters are temporally regulated and they respond differentially to stress in the form of cold shock. The precise reasons for the presence of four *rpsO*-*pnp* promoters in *S. coelicolor* are unknown but may reflect the need for *Streptomyces* to respond to changing conditions in the soil environments in which they normally grow. The *S. coelicolor* intergenic region also contains a hairpin that is involved in operon regulation (Gatewood et al., 2011). The details of that regulation will be described below in the section on RNase III.

PNPase is essential in *Streptomyces* (Bralley et al., 2006). Thus, it has not been possible to examine antibiotic synthesis in a *pnp* null mutant. Using a different approach, *pnp* was overexpressed in *S. antibioticus* leading to a 2-3-fold higher level of PNPase in the overexpression strains than in the parental strain (Bralley and Jones, 2003). Actinomycin levels, however, were lower in strains overexpressing PNPase and the levels in those strains were comparable to those observed in control strains containing only the overexpression vector. Thus, it was not possible in these studies to discern a specific effect of *pnp* overexpression on actinomycin biosynthesis. There are other studies, involving ppGpp, that bear on the role of PNPase in antibiotic production.

ppGpp is an alarmone that mediates the stringent response to amino acid starvation in bacteria (Cashel et al., 1996). The stringent response has been shown to occur in *Streptomyces* (Strauch et al., 1991). Moreover, ppGpp plays an important role in antibiotic biosynthesis. In *Streptomyces*, ppGpp production begins during vegetative growth of mycelium, increases during the transition phase prior to the onset of antibiotic production, reaches a maximum during stationary phase, when antibiotics are actively produced and declines during the later stages of stationary phase (Ochi, 1987; Kelly et al., 1991; Takano et al., 1992; Hoyt and Jones, 1999). Despite the decline during the late stages of stationary phase, ppGpp levels do not fall to zero.

In *E. coli* and other bacteria, including *Streptomyces*, ppGpp is synthesized by the RelA protein from GTP and ATP (Cashel et al., 1996). RelA interacts with ribosomes during the process of ppGpp synthesis *in vivo* and that interaction involves ribosomal protein L11 (Yang and Ishiguro, 2001). Thus, *relA* null mutations and mutations in L11 (*relC* mutations) decrease or abolish ppGpp synthesis (Cashel et al., 1996). It has been observed in several *Streptomyces* species, producing different antibiotics, that *relA* and *relC* mutations decrease or abolish antibiotic production. Thus, a *relA* mutation decreased act and red production in *S. coelicolor* (Chakraburtty and Bibb, 1997), *relA* and *relC* mutations abolished actinomycin synthesis in *S. antibioticus* (Kelly et al., 1991; Hoyt and Jones, 1999) and a *relC* mutation decreased the production of streptomycin by *Streptomyces griseus* (Ochi, 1987).

ppGpp has also been shown to affect the half-life of bulk mRNA in *S. coelicolor*. These experiments involved an *S. coelicolor* parental strain and a strain containing an inducible *relA* gene (Gatewood and Jones, 2010). The half-life of bulk mRNA was almost twice as long in the *S. coelicolor* parental strain during stationary phase as in exponential phase (5.7 min vs. 3.2 min). In the strain containing an inducible *relA* gene, producing increased levels of (p)ppGpp, induction occasioned a *ca.* two-fold increase in the half-life of bulk mRNA in stationary phase, from 6.6 to 11.8 min. No such changes in mRNA half-life were observed in a parental *E. coli* strain or its corresponding *relA* null mutant (Gatewood and Jones, 2010). Taken together, these observations suggest that (p)ppGpp may stabilize mRNAs in stationary phase *S. coelicolor* cells, as compared with cells growing exponentially.

How do these observations relate to the impact of PNPase on antibiotic production? ppGpp has been shown to inhibit both phosphorolysis and polymerization by *S. coelicolor* and *S. antibioticus* PNPases *in vitro* (Gatewood and Jones, 2010). No such inhibition was observed with PNPase from *E. coli*. Moreover, ppGpp inhibits both phosphorolysis and polymerization by PNPase from the rare actinomycete, *Nonomuraea* sp. ATCC 39727, a producer of A40926, a glycopeptide antibiotic (Siculella et al., 2010). Siculella et al. measured the half-lives of the mRNA encoding a CSR, *dpgA*, in the A40926 gene cluster in the parental *Nonomuraea* strain and in a *relC* mutant strain, producing decreased levels of ppGpp and of the glycopeptide antibiotic, A40926. Siculella et al. observed a 1.5-2-fold longer half-life (13–18 min) for *dpgA* in stationary phase in the parental strain, containing ppGpp, as compared with the *relC* mutant, with lower levels of ppGpp (9 min). These observations and those obtained in the studies of *S. antibioticus* and *S. coelicolor* suggest that the effects of ppGpp on the activity of PNPase may be involved in the stabilization of mRNAs during stationary phase and thus, in the control of antibiotic production in *Streptomyces* and other actinomycetes.

It is well established that although levels of RNA and protein synthesis decrease dramatically as *Streptomyces* cultures move from the exponential to the stationary phase of growth, a basal level of synthesis is maintained throughout stationary phase (Jones, 1976; Liras et al., 1977). This basal level of macromolecular synthesis is presumably required to produce enzymes and other proteins involved in the synthesis of the secondary metabolites, such as antibiotics, that these organisms produce in stationary phase. Stabilization of the transcripts for these proteins would represent one strategy the organisms could employ to ensure the persistence of macromolecular synthesis to support secondary metabolite production. It is known that (p)ppGpp is present in significant amounts, even in stationary phase streptomycete cultures (Takano et al., 1992; Hoyt and Jones, 1999). Thus, the inhibition of PNPase by ppGpp might represent a strategy used by actinomycetes to stabilize essential mRNAs during stationary phase, ensuring the continued production of necessary antibiotics. It would be interesting to determine whether (p)ppGpp inhibits the activity of other *Streptomyces* exo- and endonucleases.

## RNase III

Ribosomal, messenger and transfer RNAs all contain regions of secondary structure. It is not surprising, then, that ribonucleases exist that recognize and cleave in these regions. One such enzyme is RNase III, a double-strand specific endoribonuclease that is found in bacteria and eukaryotes (Nicholson, 1999; Drider and Condon, 2004). *S. coelicolor* RNase III was characterized by Chang et al. and the enzyme was shown to cleave synthetic double stranded polyribonucleotides with a preference for poly(G)-poly(C) over poly(A)-poly(U) (Chang et al., 2005). This preference is not surprising given the relatively high GC content of streptomycete genomes (Frontali et al., 1965). *S. coelicolor* RNase III also cleaves the readthrough transcript from the *rpsO*-*pnp* operon (Chang et al., 2005). However, the cleavage sites for the *S. coelicolor* enzyme differ from those of *E. coli* RNase III (Régnier and Portier, 1986). *S. coelicolor* cleaves on the two sides of a [4/4] internal loop in the *rpsO*-*pnp* intergenic hairpin. The primary cleavage site is on the 5′-side of that loop and the secondary site is on the 3′-side. In contrast, *E. coli* RNase III cleaves the intergenic hairpin within a base-paired stem situated just below an internal loop in the hairpin (Régnier and Portier, 1986). The internal loop in the *E. coli* intergenic hairpin is larger than that from the *S. coelicolor* hairpin and Calin-Jagerman and Nicholson have suggested that the features of internal loops determine the specificity of RNase III cleavage (Calin-Jageman and Nicholson, 2003).

As is also true for *E. coli* (Régnier and Portier, 1986), RNase III cleavage of the intergenic hairpin plays an important role in the regulation of expression of the *rpsO*-*pnp* operon in *S. coelicolor*. RNase III is not essential in *S. coelicolor* (or in *E. coli*) and it was possible to construct an RNase III (*rnc*) null mutant in that organism (Gravenbeek and Jones, 2008). In subsequent experiments, PNPase activity was measured and 2-4-fold higher activity levels were observed in the mutant strain during all phases of growth as compared with the parental strain (Gatewood et al., 2011). These increases correlated with increased stabilities of the transcripts derived from the *rpsO*-*pnp* operon. Thus, the half-life of the *rpsO*-*pnp* readthrough transcript increased from <<4 min (at the 4 min sampling time point, the readthrough transcript was virtually undetectable) in the parental strain to *ca.* 7 min in the null mutant, the half-life of the *pnp* transcript increased from <<4 min to 3.3 min and the *rpsO* transcript increased in half-life from *ca.* 4 min to 7 min (Gatewood et al., 2011). These results indicate that the increased PNPase activity observed in the *S. coelicolor rnc* null mutant was due to the stabilization of the mRNAs that are transcribed to produce the enzyme, *viz.* the readthrough and *pnp* transcripts. Unlike the situation in *E. coli*, the *S. coelicolor pnp* open reading frame also contained a cleavage site for RNase III (Gatewood et al., 2011). Thus, in the parental *S. coelicolor* strain, RNase III cleavage produces fragments of the transcripts from the *rpsO*-*pnp* operon with 3′-ends that can be subsequently digested by PNPase itself. RNase III thus contributes to the autoregulation of *pnp* expression in *S. coelicolor*, as it does in *E. coli* (Régnier and Portier, 1986).

RNase III also autoregulates its own expression in *S. coelicolor*. Xu et al. (2008) demonstrated increased levels of a polycistronic transcript containing *rnc* in a strain with a point mutation in *rnc* (the C120 mutation, see further below) as compared with the parental strain. They also showed that the half-life of the polycistronic transcript increased in the point mutant as compared with the parental strain. The authors synthesized the polycistronic *rnc* transcript *in vitro* and treated that transcript with isolated RNase III or with the protein containing the point mutation. The wild type protein cleaved the synthetic transcript at two positions while the mutant protein was only weakly active (but not completely inactive) against this substrate. Xu et al. used primer extension to identify the cleavage sites for RNase III in the polycistronic transcript. One of the cleavage sites was situated in the *rnc* cistron whereas the second was situated upstream of *rnc*. Both cleavages occurred in the loop regions of stem-loop structures (Xu et al., 2008).

In other studies, using an RNA Seq approach, coupled with RNA immunoprecipitation, Gatewood et al. (2012) identified *ca.* 800 transcripts from *S. coelicolor* that were bound by RNase III and therefore, were potential targets for RNase III cleavage. That number represents *ca.* 10% of the genes in the *S. coelicolor* genome (Bentley et al., 2002). By way of comparison, and using a different approach and a much smaller experimental sample size, Gitelman and Apirion identified 21 of 80 *E. coli* proteins whose abundance was affected by a point mutation in *rnc* (Gitelman and Apirion, 1980). It is noteworthy that Setinova et al. (2017) demonstrated the association of cognate antisense RNAs with 17 of the mRNAs identified in the RNA Seq experiments described by Gatewood et al.

The genetic locus encoding RNase III in *S. coelicolor* was first identified by virtue of the effect of a mutation in that locus on antibiotic production. Using N-methyl-N-nitro-N-nitrosoguanidine mutagenesis, Adamidis and Champness isolated a mutant, designated C120, that was defective in the production of all four antibiotics normally produced by *S. coelicolor* under laboratory conditions (Adamidis and Champness, 1992; Aceti and Champness, 1998). They designated this mutant *absB* (for antibiotic synthesis deficient). Act production by the mutant was reduced to only 2% of that observed in the parental strain and red production was reduced to 15% of normal levels. Cda and mmy production were also reduced (Adamidis and Champness, 1992). They subsequently showed that *actII-orf4* and *redD* levels and the level of the transcript for an act biosynthetic enzyme, *actVI-orf1*, were substantially reduced in the mutant. Huang et al. demonstrated reduced levels of the CSRs for act (*actII-orf4*), red (*redD* and *redZ*) and cda (*cdaR*) in C120 in a microarray analysis of the *S. coelicolor* transcriptome (Huang et al., 2005).

In a later study, Price et al. (1999) complemented the *absB* mutation with cloned DNA from *S. coelicolor* and showed that the *absB* gene was that for *S. coelicolor* RNase III. The C120 mutation in the RNase III gene (*rnc*) was identified as a point mutation, T188P. Price et al. constructed an *rnc* disruptant and demonstrated even lower levels of antibiotic production in that mutant as compared with C120. C120 was shown to be defective in ribosomal RNA processing, as demonstrated by the accumulation of the 30S rRNA precursor of 16S and 23S rRNAs in the mutant (Price et al., 1999).

Another level of *rnc* regulation in *Streptomyces* involves the regulatory protein, AdpA, which responds to the bacterial hormone, A-factor, a C13-γ-butyrolactone (Horinouchi and Beppu, 2007). AdpA was identified in *S. coelicolor* by Takano et al. (2003). Xu et al. (2010) demonstrated that the *adpA* transcript was cleaved by RNase III *in vitro* and *in vivo*, implicating the enzyme in the regulation of AdpA levels in *S. coelicolor*. Moreover, Xu et al. demonstrated that despite the presence of elevated levels of the mutant *rnc* transcript in the C120 *rnc* point mutant (Adamidis and Champness, 1992), the levels of the mutant RNase III protein actually decreased at later times during the C120 growth cycle. This decrease was partially reversed by the presence of an *adpA* null mutation in the C120 strain. The authors also observed a decreased expression of genes for various *S. coelicolor* proteases in the *adpA* null mutant, and argued that the lower level of mutant RNase III protein in C120 was due to the action of proteases on the mutant RNase III protein, proteases whose expression was at least partially controlled by *adpA*. Thus, the authors argue that *rnc* and *adpA* are components of a feedback loop, in which each gene product regulates the abundance of the other (Xu et al., 2010).

Xu et al. also noted precocious production of act and red in the *adpA* null mutant. The authors argued that this observation indicates that the normal function of RNase III in the control of antibiotic production in *S. coelicolor* is not dependent on AdpA. To support that point, Xu et al. (2010) demonstrated that act and red production were not restored by an *adpA*/*rnc* double mutant.

Several hypotheses have been advanced to explain the effects of an endoribonuclease, like RNase III, on antibiotic production in *S. coelicolor*. The most straightforward hypothesis would posit that RNase III is required for the processing or degradation of the transcript for an activator or repressor of antibiotic synthesis. In this regard, it is not known whether the transcripts for any of the CSRs required for antibiotic production in *S. coelicolor* are cleaved by RNase III. Price et al. suggested the possibility that RNase III itself might function as a global regulator of antibiotic production by binding to regulatory transcripts without RNA processing or degradation, thus affecting the expression of genes that are critical for antibiotic production (Price et al., 1999). There is some evidence for the regulation of gene expression in *E. coli via* the binding of RNase III to dsRNA targets without cleaving them (Oppenheim et al., 1993; Dasgupta et al., 1998).

To examine this hypothesis, Gravenbeek and Jones (2008) constructed an *rnc* null mutant in *S. coelicolor* and demonstrated that this mutant was unable to produce act and red. These authors also constructed a point mutant of *S. coelicolor rnc* that was unable to cleave a transcript that was readily cleaved by the wild type enzyme, *viz.* the *rpsO*-*pnp* readthrough transcript. This mutant, D70A, nevertheless retained the ability to bind the *rpsO*-*pnp* transcript. Gravenbeek and Jones observed that D70A could not restore act and red production to the *S. coelicolor rnc* null mutant, whereas wild type *rnc* restored full levels of antibiotic production. The authors concluded that dsRNA binding alone was insufficient to control antibiotic production in *S. coelicolor* and that the endoribonuclease activity of RNase III was required for this function.

Based on the observation that *rnc* null mutations affected ribosomal RNA processing, Sello and Buttner suggested that defective ribosomes might be formed in the null mutants and that those ribosomes could be unable to translate the long polycistronic transcripts that are characteristic of *S. coelicolor* antibiotic gene clusters (Sello and Buttner, 2008a). To examine this possibility, Gatewood and Jones (2011) cloned an operon derived from the act cluster of *S. coelicolor*, producing a transcript of *ca.* 5,700 bases in length. A kanamycin resistance gene was placed at the 3′-end of the cloned operon, replacing the normal 3′-gene, and that construct was then placed in a reporter plasmid which would allow transcription and translation of the cloned operon. The authors observed the expression of kanamycin resistance when the plasmid construct was introduced into *S. coelicolor*, indicating that the act cluster operon was being transcribed and translated in the cloning host. In addition to kanamycin resistance, Gatewood and Jones (2011) demonstrated the presence of aminoglycoside phosphotransferase, the enzyme that confers kanamycin resistance. The data support the conclusion that ribosomes in the *rnc* null mutant could translate a long polycistronic *S. coelicolor* mRNA.

Thus, two proposed mechanisms for the effects of RNase III on antibiotic synthesis in *S. coelicolor* have been eliminated and it has been demonstrated that the endoribonuclease activity of the enzyme is required for its function in antibiotic synthesis. Other possible mechanisms will be considered below. It should be noted that RNase III is required for actinomycin production in *S. antibioticus* (Lee et al., 2013) and for jadomycin production in *S. venezuelae* (Jones et al., 2014).

## Oligoribonuclease

While exoribonucleases release mononucleotides from digested substrates, endoribonucleases release oligonucleotides and larger fragments. Thus, an additional activity is required to convert oligoribonucleotides to mononucleotides which can be utilized in RNA synthesis. Oligoribonuclease possesses such an activity. The enzyme was identified in *E. coli* some years ago (Datta and Niyogi, 1975) and has subsequently been found in bacteria and eukaryotes (Zhang et al., 1998). The enzyme releases mononucleotides from the 3′-end of oligoribonucleotides 2–8 residues in length (Datta and Niyogi, 1975).

Ohnishi et al. characterized oligoribonuclease in *Streptomyces* (Ohnishi et al., 2000). These workers identified the enzyme initially in the streptomycin producer, *S. griseus*, and designated the enzyme OrnA. OrnA was shown to possess 3′-5′- exoribonuclease activity against a synthetic substrate, and its gene, *ornA*, was shown to lie just downstream of *adpA* in *S. griseus*. This suggested the possibility that *adpA* and *ornA* might be transcriptionally coupled and Ohnishi et al. demonstrated that *adpA* and *ornA* were, indeed, cotranscribed from a promoter upstream of *adpA* (Ohnishi et al., 2000).

An *ornA* null mutant grew slowly and produced sparse amounts of aerial mycelium and spores. The null mutant also produced reduced amounts of streptomycin as compared with the parental strain but the authors attributed this effect to the lower growth rate, concluding that *ornA* has no direct impact on streptomycin production in *S. griseus* (Ohnishi et al., 2000). Ohnishi et al. also demonstrated the presence of *ornA* in *S. coelicolor* and constructed a null mutant in that organism. As with *S. griseus*, the *S. coelicolor* null mutant grew slowly and formed sparse aerial mycelium, but there was essentially no effect of the null mutation on the production of act and red (Ohnishi et al., 2000).

Sello and Buttner also constructed an *ornA* null mutation in *S. coelicolor* (Sello and Buttner, 2008b). In contrast to the observations of Ohnishi et al., these workers reported that the null mutant underwent morphological differentiation and sporulation, at least on minimal medium, although more poorly than in the parental strain. The null mutant also overproduced actinorhodin. As in *S. griseus*, *ornA* is situated just downstream of *adpA* in *S. coelicolor*. Sello and Buttner performed a transcriptional analysis of *ornA* and concluded that, unlike the situation in *S. griseus*, *ornA* in *S. coelicolor* was not cotranscribed with *adpA*. This conclusion has been challenged by Xu et al. (2010) who performed RT-PCR experiments which revealed an apparent readthrough transcript in *S. coelicolor*, originating upstream of *adpA* and which contained both *adpA* and *ornA* sequences. The reason for the discrepancy between the results of Sello and Buttner and those of Xu et al. is not known at this time.

## Possible mechanisms for the function of RNases in antibiotic synthesis

Any mechanisms to explain the effects of RNases on antibiotic production in *Streptomyces* must account for at least three critical observations: (1) a null mutation in a gene encoding an RNase can affect the production of multiple antibiotics simultaneously; (2) null mutations can lead to an increase or decrease in antibiotic production; (3) the effect of an RNase on antibiotic production can apparently be achieved without affecting the CSRs in the relevant pathways.

**Figure 3:**
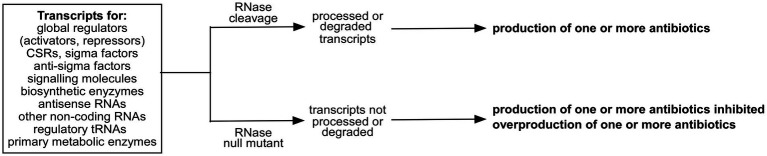
Proposed mechanism for the action of RNases on the transcripts of the various regulatory factors that have been shown to be involved in the regulation and production of antibiotics in *Streptomyces*. The model proposes that any of the transcript classes shown might be targets for RNase processing or degradation and that the effects of the RNases would then impact antibiotic production.

Given that the nuclease activities of the *Streptomyces* RNases are likely to be required for their effect on antibiotic production, a straightforward but comprehensive mechanism for those effects is presented in Figure 3. Figure 3 posits global regulators as one class of targets for *Streptomyces* RNases. In the case of RNase III for example, there may a single global regulator of act, red, cda and mmy production, whose transcript requires RNase III processing or degradation. That regulator, if it exists, remains elusive. Alternatively, it is possible that RNase III is involved in the processing of four *different* transcripts, each of which is required to produce one of the four *S. coelicolor* antibiotics. In similar fashion, any of the RNases discussed above might cleave the transcripts from any of the classes depicted in Figure 3, leading to inhibition or overproduction of antibiotics. It seems highly likely, although it is yet unproven, that RNase action on transcripts other than CSRs and other known regulators affects *Streptomyces* antibiotic production.

The field awaits the development of strategies to globally identify those transcripts.

## Author contributions

The author confirms being the sole contributor of this work and has approved it for publication.

## Conflict of interest

The author declares that the research was conducted in the absence of any commercial or financial relationships that could be construed as a potential conflict of interest.

## Publisher’s note

All claims expressed in this article are solely those of the authors and do not necessarily represent those of their affiliated organizations, or those of the publisher, the editors and the reviewers. Any product that may be evaluated in this article, or claim that may be made by its manufacturer, is not guaranteed or endorsed by the publisher.
